# The Spectrum of Vessel Wall Imaging (VWI) Findings in COVID-19-Associated Neurological Syndromes: A Review

**DOI:** 10.7759/cureus.37296

**Published:** 2023-04-08

**Authors:** Mohamad Syafeeq Faeez Md Noh, Anna Misyail Abdul Rashid, Norzaini Rose Mohd Zain

**Affiliations:** 1 Department of Radiology, Universiti Putra Malaysia Teaching Hospital, Serdang, MYS; 2 Department of Radiology, Faculty of Medicine and Health Sciences, Universiti Putra Malaysia, Serdang, MYS; 3 Department of Neurology, Faculty of Medicine and Health Sciences, Universiti Putra Malaysia, Serdang, MYS; 4 Department of Radiology, Hospital Kuala Lumpur, Kuala Lumpur, MYS

**Keywords:** neurological manifestations, neurology, vessel wall imaging (vwi), magnetic resonance imaging (mri), covid-19

## Abstract

Since the start of the pandemic, there have been extensive studies from all over the world reporting on coronavirus disease 2019 (COVID-19)-associated neurological syndromes. Although initially thought of as primarily a respiratory pathogen, it became increasingly clear that the virus does have other systemic manifestations, including on the neurological system. Since then, the discovery of the many neuroimaging features of COVID-19-associated neurological syndromes have puzzled researchers and physicians in terms of interpretation, and how best to manage these findings to benefit patients. We sought to review the neuroimaging findings of COVID-19-associated neurological syndromes, particularly the vessel wall imaging (VWI) features, in the hope of finding a common feature that would better guide physicians in terms of further management of this group of patients. We will also look into the potential pitfalls of interpreting the VWI findings in these patients.

## Introduction and background

Coronavirus disease 2019 (COVID-19)-associated neurological syndromes have been extensively reported worldwide [1-3]. Among the many hypotheses as to why these patients manifest neurological signs and symptoms is attributable to an underlying inflammatory or immune mechanism. Neuropathologic studies have demonstrated the presence of vascular inflammation affecting the endothelium in these patients [4,5]. It is with this in mind that advanced imaging to better interrogate the vessel wall becomes a necessity - hence the need for vessel wall imaging (VWI). However, up to this day, there are limited studies describing the VWI findings in these patients. We sought to look at the VWI findings reported by these studies, with the intention to look at the most common features that would hopefully assist in terms of planning the imaging sequence, anticipating the imaging findings, and further management of affected patients.

## Review

Material and methods

In order to identify relevant studies, we searched the PubMed, Google Scholar, Web of Science, and Scopus databases using search terms including “COVID-19”, “coronavirus disease 2019”, “neurology”, “COVID-19 neurology”, “MRI”, “Magnetic resonance imaging”, “Vessel wall imaging”, “VWI” and combinations of these terms. We looked at full-text papers in the English language published from January 2020 until July 2022. Articles that described the magnetic resonance imaging (MRI) findings but did not mention the VWI findings were excluded, as these were beyond the scope of this paper. We also examined the reference lists of key papers to identify further articles that are potentially relevant for inclusion in this review.

Results 

After performing an extensive literature search and excluding irrelevant articles, we found a total of eight papers [6-13] that described the VWI findings in patients with COVID-19-associated neurological syndromes. All of the authors read the articles and wherever possible, extracted the following information - the demographics, days from COVID-19 respiratory symptom onset to MRI, neurological history, risk factors for vascular disease, neurological presentation, cerebrospinal fluid (CSF) findings if any, the MRI/neuroimaging findings in general, as well as the description of the VWI findings. The above-mentioned details are arranged into a table, when present. When absent, these details are summarized, and can be referred to the original publication. Cumulatively, across all eight papers, a total of 114 patients had VWI done as part of the clinical workup. Of these 114 patients, 45 patients (39.5%) with positive VWI findings were found. However, when looking at the percentage of positive VWI findings for each individual study, the percentage of patients with positive findings ranged from 16% (lowest) to 100% (highest); it is important to keep in mind that the smaller the cohort, the higher the chances of the percentage being higher. These extracted data are discussed briefly below, and summarized in Table 1 and Table 2. 

**Table 1:** Summary of the studies, publication years, the number of patients who underwent VWI, the number of patients with positive VWI findings, as well as the percentage for each individual study. VWI = vessel wall imaging

No.	Study authors	Publication year	Number of patients who had VWI done	Number of patients with positive VWI findings (percentage)
1	Kremer et al [6]	2021	69 patients	11 patients (16%)
2	Lovblad et al [7]	2021	34 patients	24 patients (71%)
3	Keller et al [8]	2020	3 patients	3 patients (100%)
4	Mazzacane et al [9]	2022	4 patients	3 patients (75%)
5	Callen et al [10]	2021	1 patient	1 patient (100%)
6	Dixon et al [11]	2021	1 patient	1 patient (100%)
7	Gulko et al [12]	2020	1 patient	1 patient (100%)
8	Md Noh et al [13]	2022	1 patient	1 patient (100%)

**Table 2:** Summary of the demographics, clinical history and symptoms, VWI and other relevant neuroimaging findings, as well as the CSF findings when present for each individual patient from the included studies when available. Individual patient details from the studies by Keller et al. [8] and Lovblad et al. [7] are not available; the summarized findings can be referred to the original publications. MRI = magnetic resonance imaging, VWI = vessel wall imaging, AIS = acute ischemic stroke, CSF = cerebrospinal fluid, MCA = middle cerebral artery, PCA = posterior cerebral artery, ACA = anterior cerebral artery, ICA = internal carotid artery, WM = white matter, FLAIR = fluid attenuated inversion recovery, GCS = Glasgow Coma Score

Study authors/patient no.	Sex	Age	Days from COVID-19 respiratory symptoms onset to brain MRI	Neurological history	Risk factors for vascular disease	Neurological manifestation	VWI findings/localization of vessel wall enhancement	Other neuroimaging findings	CSF findings
Kremer et al, 2021 [6] Patient 1	Male	69	34	-	Hypertension, Diabetes mellitus type 2, Hyperlipidemia, Obesity	Pathological wakefulness after sedation	Basilar artery, left MCA, bilateral PCA	AIS in anterior choroidal artery territory, extensive WM microhemorrhages	Not available
Patient 2	Male	70	36	-	-	Pathological wakefulness after sedation/right pyramidal syndrome	Left MCA	Extensive WM microhemorrhages, subarachnoid hemorrhages	10 cells/mm3, raised total protein 0.75 g/L, raised IgG 123 mg/L, oligoclonal bands identical in CSF and serum, SARS-CoV-2 ARN-
Patient 3	Male	79	40	-	Hyperlipidemia	Pathological wakefulness after sedation	Basilar artery, bilateral MCA	Extensive WM microhemorrhages, subarachnoid hemorrhages	3 cells/mm3, total protein 0.4 g/L, raised IgG 47 mg/L, oligoclonal bands identical in CSF and serum, SARS-CoV-2 ARN-
Patient 4	Male	75	36	-	-	Pathological wakefulness after sedation, right clonic seizures	Basilar artery, left MCA	Extensive WM microhemorrhages	Not available
Patient 5	Male	61	34	-	Hyperlipidemia, smoking	Pathological wakefulness after sedation	Basilar artery, bilateral MCA	Extensive WM microhemorrhages	0 cell/mm3, total protein 0.4 g/L, IgG 27 mg/L, oligoclonal bands identical in CSF and serum, SARS-CoV-2 ARN-
Patient 6	Male	66	12	-	Hyperlipidemia, smoking	Confusion/agitation	Basilar artery	-	0 cell/mm3, raised total protein 0.6 g/L, raised IgG 51 mg/L, SARS-CoV-2 ARN-
Patient 7	Male	67	34	Transient ischemic attack	Hypertension, hyperlipidemia	Impaired consciousness, agitation	Basilar artery, bilateral MCA, bilateral PCA	-	Not available
Patient 8	Male	64	21	-	Hypertension, type 2 diabetes mellitus	Bilateral pyramidal syndrome, aphasia, confusion	Basilar artery, bilateral MCA	AIS (Left ACA-MCA watershed cerebral infarction), FLAIR hyperintense lesions involving both middle cerebellar peduncles	0 cell/mm3, raised total protein 0.71 g/L, raised IgG 60 mg/L, oligoclonal bands identical in CSF and serum, SARS-CoV-2 ARN-
Patient 9	Female	71	32	-	Hypertension, type 2 diabetes mellitus, hyperlipidemia, obesity	Pathological wakefulness after sedation	Bilateral MCA and PCA	Subarachnoid hemorrhages, extensive and confluent supratentorial white matter FLAIR hyperintensities	2 cells/mm3, total protein 0.3 g/L, raised IgG 42 mg/L, oligoclonal bands identical in CSF and serum, SARS-CoV-2 ARN
Patient 10	Male	65	22	Transient ischemic attack	Hypertension, type 2 diabetes mellitus	Pathological wakefulness after sedation	Basilar artery	AIS (bilateral ACA-MCA and MCA-PCA watershed cerebral infarction)	Not available
Patient 11	Male	73	37	-	-	Pathological wakefulness after sedation, bilateral pyramidal syndrome	Bilateral MCA and PCA	AIS (bilateral ACA-MCA and MCA-PCA watershed cerebral infarction), extensive WM microhemorrhages	Not available
Callen et al, 2021 [10] Patient 1	Female	51	8	1-day history of altered mentation and gait instability	Diabetes mellitus, hypertension	Mild encephalopathy, moderate right-sided hemiparesis	Long segment concentric enhancement of the left MCA	Subacute infarcts at the left MCA distribution	0 cells/mm3, normal glucose 55 mg/dL, normal protein 30 mg /dL, normal IgG index 1090 mg/dL, 0 unique oligoclonal bands, no SARS-CoV-2 RNA detected
Dixon et al, 2021 [11] Patient 1	Male	64	29	-	Diabetes mellitus, hypertension, hyperlipidemia, ischemic heart disease	Low GCS, episodic clonic movements of the proximal upper limbs on sedation hold	Long segment, abnormal concentric vessel wall enhancement of both MCA, ACA, vertebral arteries, and basilar artery	Multiple subacute infarcts at the right MCA and both PCA territories	Not performed
Gulko et al, 2020 [12] Patient 1	Female	13	2 months	Fluctuating but persistent headache, speech difficulties, right upper and lower extremities weakness for 4 days	No known medical illness	Dysfluent speech, word-finding difficulties, mild to moderate extensor weakness in the right arm and leg	Wall thickening and marked concentric enhancement at stenosis site (M1 segment of the left MCA)	Subacute infarct involving the left MCA territory	Normal
Md Noh et al, 2022 [13] Patient 1	Male	60	10	Right-sided body weakness, unable to follow command, confusion	Hypertension, dyslipidemia	Right-sided dense hemiparesis, right hemisensory loss, upgoing plantar reflex, no gaze preference or hemianopia	Circumferential vessel wall enhancement of the left ACA and ICA	Acute left internal watershed infarct, left ACA territory infarct	Not done
Mazzacane et al, 2021 [9] Patient 1	Male	67	At onset	-	Hypertension	-	Focal concentric enhancement at A2 segment of ACA	Bilateral acute ischemic lesions at ACA territory	Normal
Patient 2	Male	56	7	-	Hypertension	-	Focal concentric enhancement at M1-M2 segment right MCA	Acute ischemic lesions at right MCA territory, subacute left frontal ischemic lesion	Not performed
Patient 3	Male	58	21	-	Diabetes mellitus, hypertension	-	Multifocal enhancement	Bilateral acute ischemic lesions	Oligoclonal bands in CSF and serum, mirror pattern

Review of included studies

Demographics 

Of the six studies where the individual patient data are available to be extracted, a total of 18 patients were included, where the majority were males (n=15) as opposed to females. The age of the patients ranged from 13 years old (the youngest and only pediatric patient in the cohort) to 79 years old, with a mean age of 62.7. The summarized demographics from the studies by Keller et al. [8] and Lovblad et al. [7] can be referred to the original publications. *Neurological History and Manifestations*

In the majority of patients with positive VWI findings, the two main neurological histories and manifestations were stroke-related; these ranged from transient ischemic attacks to unilateral weakness/hemiparesis, as well as encephalopathy. However, as a considerable portion of these patients also presented with severe COVID-19 pneumonia requiring ventilation, they were also found to have pathological wakefulness after sedation, which prompted neuroimaging for further assessment. Other neurological symptoms included altered mentation/Glasgow Coma Score (GCS), confusion, agitation, abnormal movements, aphasia, gait instability, and bilateral pyramidal syndrome. *Vessel Wall Imaging (VWI) Findings *

Of the 45 patients across the eight studies who had VWI done, 44 patients (98%) had circumferential, concentric vessel wall enhancement of the involved vessels, either with or without narrowing. These were, for the most part, long segmented. Only one patient from the study by Mazzacane et al. [9] had multifocal enhancement. The pattern of vessel wall enhancement mimics those seen in patients with cerebral vasculitis. *Other MRI/Neuroimaging Findings *

The majority of patients had ischemic lesions involving the territories supplied by the pathologically enhanced vessels. However, a portion of these patients also demonstrated cerebral microhemorrhages, subarachnoid hemorrhages (presumably non-aneurysmal, as these were not mentioned), as well as white matter hyperintensities. *Cerebrospinal Fluid (CSF) Findings *

The data regarding the CSF findings from the included studies are heterogenous, with some studies having no available data for the CSF findings. However, it would be interesting to note that in the studies that do, the SARS-CoV-2 in the CSF were all negative. The summary of the CSF findings for available individual patients can be referred to in Table 2. The summarized findings from the studies by Keller et al. [8] and Lovblad et al. [7] can be referred to in the original publications. The detailed analysis and discussion of the CSF findings is beyond the scope of this paper.

Discussion

The main aim of this review was to look at the spectrum and possible common pattern of intracranial VWI findings in patients with COVID-19-associated neurological syndromes, across all published papers thus far. Based on our review, we have found that in most patients, the pattern of vessel wall imaging is akin to those seen in patients with cerebral vasculitis. Based on the expert consensus recommendations of the American Society of Neuroradiology (ASNR), a vasculitis is suspected when the vessel wall imaging pattern is homogenous and concentric, with associated wall thickening [14]. When vessel wall enhancements are non-concentric, the possibility of an atherosclerotic plaque needs to be considered. This is especially important to consider, because presence of atherosclerotic plaques may be associated with the development of vasa vasorum [15], a condition that may mimic vasculitis. A possible explanation for this pattern of vessel wall thickening and enhancement is the pathophysiological mechanism by which COVID-19 attacks the blood vessels. The main receptor for SARS-CoV-2 is angiotensin converting enzyme 2 (ACE-2), which facilitates the entry of the virus and can be found among other human cell surfaces within the endothelium, leading to endotheliitis. This theory is further supported by neuropathologic studies, which have demonstrated the presence of vascular inflammation affecting the endothelium in these patients [4,5]. Apart from concentric, diffuse, vessel wall thickening and enhancement, other neuroimaging findings noted were ischemic lesions within the involved vascular territories, subarachnoid hemorrhages, cerebral hemorrhages, and white matter hyperintense lesions - which could all well be associated complications from cerebral vasculitis. Additionally, some of these patients also demonstrated cerebral microbleeds, which in some atypically involved the corpus callosum. This atypical involvement has been described in critically ill COVID-19 patients [16,17]. However, the pathophysiological mechanism bringing about this finding remains unknown. The theories that have been proposed include those related to hypoxemia as well as vasculitis. Cerebral microbleeds have also been noted in COVID-19 patients presenting with neuroimaging findings of leukoencephalopathy; in severe cases this is termed acute hemorrhagic leukoencephalopathy (AHLE). Other possible theories include the consumption coagulopathy seen in these patients, which leads to thrombosis of the small medullary veins, consequently leading to microhemorrhages [5,18]. Due to the growing need for neuroimaging in patients with COVID-19-associated neurological syndromes, the Subspecialty Committee on Diagnostic Neuroradiology of the European Society of Neuroradiology (ESNR) recently came up with an expert consensus [19] that aims to standardize the imaging protocol in this group of patients. A basic MRI protocol of T2-weighted, fluid attenuated inversion recovery (FLAIR) (preferably 3D), and diffusion weighted images, as well as hemorrhage-sensitive sequences (preferably susceptibility weighted imaging [SWI]) is recommended. Post gadolinium 3D FLAIR images are also recommended, for the detection of leptomeningeal contrast enhancement. However, the recommendation for VWI is somewhat unclear, and is recommended to be performed only in select patients where there is a strong clinical or radiological suspicion that lesions could be secondary to a vasculitic rather than a thrombotic/embolic process. The committee however also acknowledges that vasculitis in this group of patients may be much more common than initially thought. We hope that with this review, the threshold for VWI in patients with COVID-19-associated neurological syndromes becomes lower - to the benefit of patients, as well as to facilitate physicians in better managing these patients. With regards to the neurological history and manifestations, it is worthy to note that most patients with COVID-19-associated neurological syndromes with positive VWI findings had stroke syndromes to a certain degree, as well as encephalopathy as the primary clinical manifestation. Apart from these, other neurological manifestations include altered mentation/GCS, confusion, agitation, abnormal movements, aphasia, gait instability, and bilateral pyramidal syndrome. For those with severe COVID-19 pneumonia requiring ventilation, poor GCS recovery when on sedation hold seems to be a primary feature in those with positive VWI findings. It is safe to say that although some neurological manifestations are predominant, affected patients may manifest a wide variety of neurological signs and symptoms - hence imaging with VWI needs to be considered on a case-to-case basis, within the policy of each center.

Potential pitfalls 

When subjecting these patients to VWI, it is important to note that the interpretation of the VWI findings is also reliant to the patient’s clinical presentation, location of the brain lesion, the therapeutic agents that the patient is currently being administered (patients on steroid therapy may not manifest vessel wall enhancement due to the effect of the therapy), as well as non-pathologic conditions that may mimic pathologies. For example, enhancement of the vasa vasorum or vascular plexus, which are present in extracranial arteries mainly but may also involve the intracranial segments of the vertebral arteries (proximal portion of the V4 segment) [20] is considered non-pathological. It is worthy to note that in children or healthy young adults, vasa vasorum is not typically present [15]. Another common misinterpretation of vascular enhancement as vasculitis or atherosclerotic plaque is the area surrounding a partially collapsed petrous carotid artery segment when there is ipsilateral hemodynamic impairment. This area, when enhanced, reflects a compensatory expansion of the venous plexus within the petrous canal, and not due to arterial wall thickening and enhancement, which is the very imaging definition of vasculitis [21]. 

## Conclusions

COVID-19 neurological syndromes may manifest in a multitude of presentations. In those with positive VWI findings, the most common presentations based on our review are stroke syndromes and encephalopathy, with vessel wall imaging features akin to those with cerebral vasculitis. The prevalence of cerebral vasculitic changes may be much more common than initially anticipated, hence there is a need for a much more widespread use of VWI, to better assess and manage these patients. The current available guidelines may be able to help standardize imaging in this group of patients, to better understand the pathophysiological processes and better help clinicians in patient care and management. 
